# Dietary Habits, Nutrients and Bone Mass in Spanish Premenopausal Women: The Contribution of Fish to Better Bone Health 

**DOI:** 10.3390/nu5010010

**Published:** 2012-12-27

**Authors:** Julian F. Calderon-Garcia, Jose M. Moran, Raul Roncero-Martin, Purificacion Rey-Sanchez, Francisco J. Rodriguez-Velasco, Juan D. Pedrera-Zamorano

**Affiliations:** Metabolic Bone Diseases Research Group, School of Nursing and Occupational Therapy, University of Extremadura, Caceres 10003, Spain; E-Mails: jfcalgar@unex.es (J.F.C.-G.); jmmorang@unex.es (J.M.M.); rronmar@unex.es (R.R.-M.); prey@unex.es (P.R.-S.); fcorodriguezv@unex.es (F.J.R.-V.)

**Keywords:** vitamin D, bone mass, quantitative bone ultrasound, fishes

## Abstract

The moderate consumption of fish is recommended for a healthy diet and is also a feature of the Mediterranean diet. Fish is a major food group in diets throughout the world, and studies show that fish consumption is associated with a lower risk of a number of conditions. Spain has one of the highest annual per capita consumptions of fish worldwide. As fish is a source of high quality protein; *n*-3 polyunsaturated fatty acids; vitamins, such as A and D; and minerals, such as selenium, calcium, iodine, magnesium, copper and zinc, nutrients that have positive effects on bone characteristics, it has been proposed that its consumption could improve bone health. In this cross-sectional study, we have investigated the relationship between dietary habits and nutrient intake of 151 Spanish premenopausal women and analyzed the association of fish consumption on bone mass measured by quantitative ultrasound of the phalanges. A higher (*P* < 0.05) bone mass and vitamin D intake (*P* < 0.05) was observed in the group with a fish intake of 5–7 servings/week. We conclude that increased fish consumption is helpful in maintaining an adequate bone mass in Spanish premenopausal women.

## 1. Introduction

A moderate consumption of fish is recommended for a healthy diet and is also a feature of the Mediterranean diet. Between 2004 and 2008, the EU funded an integrated research project, SEAFOODplus, which developed two transverse consumer surveys in Belgium, Denmark, the Netherlands, Poland, France and Spain. In both studies, Spain had one of the highest annual per capita consumptions of fish in Europe, with an average of 100 g/person/day, which is elevated compared to the national standard dietary guidelines [1].

Diet is an important modifiable factor in the development and maintenance of bone mass, but there are few data available on the relationship between food intake and bone health, with the exception of milk and soy products [2]. Fish is a major food group in diets throughout the world, and studies show that fish consumption is associated with a lower risk of cancer [3], combined cardiovascular disease [4] and mortality [5]. Fish is a source of high quality protein; *n*-3 polyunsaturated fatty acids (PUFAs); vitamins, such as A and D; and minerals, such as selenium, calcium, iodine and zinc [6]. Many of these nutrients have positive effects on bone health. The positive effects of calcium and vitamin D, which are the most important nutrients for bone health, have long been established. In Spain, fish consumption accounts for 87% of total dietary vitamin D intake [1]. Zinc in the diet can also have a positive correlation with bone mineral density (BMD) [7]. Recent studies have also found that PUFAs are associated with increased bone mass in humans [8,9,10]. Although a correlation between fish consumption and a higher bone mass has been reported [11,12], this association remains controversial [13]. 

It has been suggested that a holistic approach to nutrition, one that examines the effects of diet in relation to nutrients, foods or food groups in chronic disease prevention and treatment, may be the most sensible approach in studying the relationship between diet and biological markers [14]. In this cross-sectional study, we investigated the relationship between dietary habits and nutrient intake of 151 Spanish women and analyzed the association of fish consumption on bone mass.

## 2. Experimental Section

### 2.1. Subjects

A total of 151 healthy women were included in this cross-sectional study, with a mean age of 35 (SD ± 10) years and a BMI between 19 and 32 kg/m^2^. Histories indicating current and prior menstrual regularity (11–13 cycles/year) were recorded. Participants were recruited in a clinical convenience sample. All of the subjects resided in the health district of the province of Caceres, Spain. They all gave written, informed consent. The Office for Protection Against Research Risks of the University of Extremadura approved the study.

Before each candidate was enrolled in the study, a complete medical history was taken, and a physical examination was performed. Normality was also established by weight, within the range of 70%–130% of ideal body weight for height, and by the results of a biochemical study of blood glucose, transaminases, γ-glutamyl transpeptidase, creatinine, calcium, phosphorus, total protein, bilirubin, alkaline phosphatase and tartrate-resistant acid phosphatase as well as a coagulation study. In all cases, the calcium level was corrected using the protein level. A radiologic study of the thoracic and lumbar spine excluded vertebral deformities, defined as the loss of >25% of the height of the anterior, middle or posterior vertebral body, compared with normal reference values in persons matched for age and sex, as previously reported by our group [15]. The study subjects were not taking any medications and had no diseases, including those associated with abnormalities in mineral metabolism (diabetes mellitus, liver disease, renal osteodystrophy or parathyroid, thyroid, adrenal or ovarian disease) that could interfere with calcium metabolism. All subjects led active lives, but none practiced any recreational or professional sports.

Height measurements were made using a Harpender stadiometer with the mandibular plane parallel to the floor, and patients were weighed on a biomedical balance. Both measurements were made with subjects wearing pajamas but no shoes. BMI was calculated by dividing the weight in kilograms by the square of the height in meters (kg/m^2^). Ideal weight was calculated in relation to height. Food was quantified using a dietetic scale, measuring cups, cans, small bottles and spoons on the basis of current 7-day dietary records, as in previous studies [16,17]. The questionnaire used was self-reported, and the person completing the interview was blinded to the research question and hypothesis. 

### 2.2. Ultrasound Studies

As in previous studies [15,16,17], all of the women underwent an ultrasound study of the second to fifth proximal phalanges of the non-dominant hand, calculating the mean of all the measurements. The ultrasound study was made with a model DBM Sonic Bone Profiler (Igea, Capri, Italy) equipped with a calliper that closes tangentially on the phalanx and measures the amplitude-dependent speed of sound (Ad-SoS) in meters per second through the phalanx. Positioning and repositioning the instrument is easy because it uses the prominences of the lower phalangeal epiphysis as a reference; the clip is placed just behind the prominences. The instrument transmits at a frequency of 1.2 MHz with 22 W of power. The instrument precision was determined from three measurements in eight subjects at time intervals not exceeding 21 days. The coefficient of variation was 0.77%. The inter-observer coefficient of variation was 1.1%.

### 2.3. Statistical Studies

The normal distribution of the data was confirmed by calculating the skewness and kurtosis before applying standard tests. Fisher’s exact test, correlation *r* to *z* and partial correlation analyses, an unpaired *t* test and an analysis of variance (ANOVA) were used when appropriate to examine relationships between variables our rationale was to ensure the greatest possible homogeneity in the identified fish consumption subgroups. Subgroup determination was based on a clustering approach. The subjects were first classified using parallel hierarchical clustering and a k-means model. Based on this model, 4 subgroups were obtained: 0–2, 3–4, 5–7 and 8–14 servings per week. A *P* value < 0.05 was statistically significance. Step-wise multiple linear regression analysis was executed to estimate the linear relationship between dependent variable (Ad-SoS) and various independent variables. Data were processed using the StatView 5.01 statistical package (SAS Institute, Cary, NC, USA).

## 3. Results

### 3.1. General Characteristics of the Study Population

A total of 151 women participated in the study (34.94 ± 9.89 years). The BMI and standard deviation were calculated, and the mean BMI (in kg/m^2^) was 22.36 ± 1.58. Characteristics of the study sample in terms of anthropometric characteristics, nutrient intake and dietary habits are shown in Table 1.

**Table 1:** Biological, anthropometric and dietetic factors in the studied sample.

	Mean ± SD	Range	Reference ranges
Age (years)	34.94 ± 9.89	18–54	
Menarche age (years)	12.13 ± 0.84	10–13	
Gravidity	1.32 ± 1.03	0–3	
Births	1.32 ± 1.01	0–3	
Weight (kg)	58.01 ± 5.10	46–70	
Height (m)	1.61 ± 0.06	1.45–1.80	
BMI (kg/m^2^)	22.36 ± 1.58	19.14–24.92	
Ad-SoS (m/s)	2125.63 ± 49.83	1986–2324	
Vitamin D (IU/day)	252.02 ± 208.99	27.2–1356	200
Vitamin E (mg/day)	2.59 ± 0.86	1.05–5.80	8
Ca (mg/day)	880.58 ± 332.73	210–1481	800
P (mg/day)	1171.19 ± 304.3	553–2053	800
Ca/P (mg/mg)	0.74 ± 0.17	0.307–1.19	≥1
Fe (mg/day)	12.52 ± 4.2	5.42–28.86	17–21
Zn (mg/day)	8.62 ± 2.56	3.78–19.21	15
Proteins (g/day)	77 ± 19.94	37.05–152.47	47
Ca/Proteins (mg/g)	11.68 ± 4.29	3.48–26.09	≥20
Proteins/weight (g/kg)	1.34 ± 0.38	0.59–2.57	
Fats (g/day)	76.79 ± 19.70	27.94–128.33	90
Carbohydrates (g/day)	249.09 ± 74.44	86.1–410.50	330
Kcal	1986.85 ± 400.16	840.6–2582.40	
I (μg/day)	264.6 ± 215.25	6–960	140
Mg (mg/day)	230.58 ± 102.46	82–649.10	350
F (μg/day)	618.41 ± 282.39	143–2055	1500–3000
Cu (mg/day)	1.19 ± 1.33	0.30–8.41	2
Se (μg/day)	78.85 ± 32.02	22.2–176.40	55
Folic acid (μg/day)	144.45 ± 49.07	35–307.70	200
Fats (servings per week)	9.52 ± 3.50	1–25	
Dairy (servings per week)	12.6 ± 4.88	2–28	18–24
Meat (servings per week)	8.44 ± 3.39	1–20	3–4
Fish (servings per week)	4.17 ± 2.26	0–14	3–4
Cereal (servings per week)	13.01 ± 4.73	3–29	28–42
Fruit (servings per week)	10.58 ± 6.07	1–32	>21
Vegetables (servings per week)	8.63 ± 5.66	0–33	>14
Sugars (servings per week)	10.11 ± 5.31	0–28	
Eggs (servings per week)	1.97 ± 1.31	0–7	3–4
Beer (servings per week)	2.42 ± 3.41	0–18	
Wine (servings per week)	0.61 ± 1.65	0–11	

**Table 2:** Biological and anthropometric factors, distributed by fish consumption in premenopausal Spanish women.

	Fish (servings/week)							
	0–2 (*n* = 32)		3–4 (*n* = 65)		5–7 (*n* = 46)		8–14 (*n* = 8)	
	Mean (SD)	Range	Mean (SD)	Range	Mean (SD)	Range	Mean (SD)	Range
Age (years)	34.22 (10.13)	18–48	35.11 (10.26)	18–54	35.07 (9.29)	18–50	35.75 (10.89)	22–50
Menarche age (years)	12.03 (0.82)	11–13	12.15 (0.78)	10–13	12.24 (0.82)	11–13	11.63 (1.30)	10–13
Gravidities	1 (1)	0–3	2 (1)	0–3	1 (1)	0–3	2 (0)	2–2
Births	1 (1)	0–3	2 (1)	0–3	1 (1)	0–2	2 (0)	2–2
Weight (kg)	56.99 (4.44)	49.80–65	58.82 (5.12)	46.80–69	57.63 (5.47)	46–70	57.54 (5.08)	48–63
Height (m)	1.60 (0.06)	1.46–1.74	1.61 (0.06)	1.50–1.71	1.62 (0.07)	1.45–1.80	1.61 (0.05)	1.55–1.70
BMI (kg/m^2^)	22.28 (1.55)	19.32–24.92	22.62 (1.51)	19.56–24.89	22.05 (1.66)	19.15–24.80	22.30 (1.68)	19.98–24.13
Ad-SoS (m/s)	2108.84 (39.20)	2030–2178	2122.43 (52.51)	1986–2239	2139.51 (50.07)	2041–2324	2140.75 (46.86)	2070–2206

**Table 3:** Nutrient intake, distributed by fish consumption in premenopausal Spanish women.

	Fish (servings/week)							
	0–2 (*n* = 32)		3–4 (*n* = 65)		5–7 (*n* = 46)		8–14 (*n* = 8)	
	Mean (SD)	Range	Mean (SD)	Range	Mean (SD)	Range	Mean (SD)	Range
Vitamin D (IU/day)	191.30 (142.46)	(32–580.80)	215.78 (170.59)	(27.20–920)	325.84 (246.79)	(52.80–1356)	364.95 (330.21)	(82.40–1092)
Vitamin E (mg/day)	2.41 (0.91)	(1.22–5.80)	2.60 (0.85)	(1.06–5.15)	2.62 (0.87)	(1.06–4.94)	3.08 (0.45)	(2.45–3.75)
Ca (mg/day)	812.34 (372.22)	(238–1478)	874.35 (317.96)	(226–1481)	933.70 (327.68)	(210–1474)	898.75 (322.02)	(421–1332)
P (mg/day)	1067.47 (313.41)	(594–2053)	1151.31 (296.20)	(553–1786)	1241.74 (292.63)	(625–1987)	1341.88 (278.92)	(936–1669)
Ca/P (mg/mg)	0.74 (0.22)	(0.32–1.19)	0.75 (0.16)	(0.31–1.05)	0.74 (0.15)	(0.31–1.01)	0.65 (0.17)	(0.44–0.98)
Fe (mg/day)	12.30 (5.03)	(5.44–26.59)	12.21 (4.07)	(5.42–25.69)	12.90 (4)	(7.89–28.86)	13.70 (2.92)	(10.76–18.87)
Zn (mg/day)	7.63 (2.34)	(3.78–12.92)	8.48 (2.20)	(4.13–15.93)	9.10 (2.82)	(5.03–19.21)	10.97 (2.88)	(7.44–15.10)
Proteins (g/day)	67.69 (18.56)	(37.72–121.31)	75.56 (20.12)	(37.05–135.40)	81.08 (14.90)	(44.79–117.88)	102.42 (24.53)	(74.85–152.47)
Ca/Proteins (mg/g)	12.50 (5.86)	(3.77–26.09)	11.77 (3.78)	(3.48–22.15)	11.50 (3.75)	(3.55–20.88)	8.73 (2.84)	(5.38–14.28)
Proteins/weight (g/kg)	1.20 (0.35)	(0.59–2.11)	1.30 (0.39)	(0.64–2.46)	1.42 (0.31)	(0.68–2.09)	1.79 (0.41)	(1.23–2.57)
Fats (g/day)	72.40 (21.19)	(27.94–115.34)	75.91 (20.73)	(33.86–128.33)	79.44 (18.09)	(42.01–121.13)	86.20 (7.07)	(75.38–98.30)
Carbohydrates (g/day)	218.66 (71.37)	(86.10–400.20)	255.28 (66.47)	(130.30–380.40)	247.90 (79.63)	(98.20–384)	324.28 (62.45)	(217.60–410.50)
Kcal	1770.22 (417.13)	(840.60–2484)	2015.52 (374.22)	(1273.30–2577.40)	2029.78 (378.89)	(1093–2560)	2373.63 (228.79)	(2055.60–2582.40)
I (μg/day)	216.81 (187.07)	(6–681)	260.61 (217.11)	(6–705)	312.22 (238.42)	(9–960)	219.75 (125.16)	(35–346)
Mg (ng/day)	221.88 (102.37)	(82–599.20)	233.60 (104.57)	(104.90–627.80)	233.88 (107.12)	(110.30–649.10)	221.84 (64.96)	(153.80–337.20)
F (μg/day)	459.41 (251.52)	(165–1335)	615.10 (253.67)	(143.00–1389)	644.00 (187.62)	(257–1082)	1134.25 (435.84)	(508–2055)
Cu (mg/day)	1.12 (1.42)	(0.30–8.41)	1.24 (1.35)	(0.37–6.99)	1.21 (1.39)	(0.39–7.93)	1.05 (0.47)	(0.54–1.78)
Se (μg/day)	64.42 (28.93)	(22.20–127)	78.40 (30.71)	(22.90–141.90)	83.53 (28.54)	(25.80–141.30)	113.31 (44.06)	(57.10–176.40)
Folic acid (μg/day)	135.78 (52.39)	(63.60–256.30)	143.50 (47.42)	(35–307.70)	148.50 (45.47)	(75.90–279)	163.48 (68.72)	(87.70–293.60)

**Table 4:** Dietary habits, distributed by fish consumption in premenopausal Spanish women.

(servings per week)	Fish (servings/week)							
	0–2 (*n* = 32)		3–4 (*n* = 65)		5–7 (*n* = 46)		8–14 (*n* = 8)	
	Mean (SD)	Range	Mean (SD)	Range	Mean (SD)	Range	Mean (SD)	Range
Fats	8.44 (3.36)	(1–20)	9.48 (3.10)	(2–19)	9.96 (3.43)	(5–22)	11.75 (6.04)	(7–25)
Dairy	10.56 (4.99)	(2–20)	12.77 (4.05)	(3–27)	13.24 (5.35)	(2–28)	15.63 (5.80)	(5–22)
Meat	8.22 (3.87)	(1–15)	8.48 (3.27)	(2–20)	8.28 (3.19)	(1–18)	10.14 (3.67)	(4–14)
Fish	1.56 (0.72)	(0–2)	3.51 (0.50)	(3–4)	5.89 (0.82)	(5–7)	10 (2.56)	(8–14)
Cereal	12.34 (3.92)	(5–20)	12.69 (4.34)	(4–22)	13.26 (5.14)	(3–24)	16.75 (7.11)	(5–29)
Fruit	9.22 (4.95)	(1–22)	10.85 (5.61)	(2–23)	10.93 (6.44)	(2–29)	11.88 (10.76)	(1–32)
Vegetables	6 (4.59)	(0–19)	8.43 (5.60)	(0–24)	10.13 (4.99)	(2–24)	12.71 (9.52)	(4–33)
Sugars	8.97 (4.51)	(0–16)	10.20 (5.15)	(1–21)	11.11 (5.95)	(1–28)	8.25 (5.15)	(2–16)
Eggs	1.66 (1.12)	(0–5)	1.92 (1.35)	(0–7)	2.04 (1.30)	(0–6)	3.25 (1.04)	(2–4)
Beer	2.44 (3.20)	(0–15)	2.37 (3.63)	(0–16)	2.39 (3.51)	(0–18)	2.88 (2.03)	(0–7)
Wine	0.56 (2.02)	(0–11)	0.32 (1.03)	(0–6)	0.80 (1.72)	(0–9)	2 (2.88)	(0–7)

### 3.2. Anthropometric Characteristics, Nutrient Intake and Dietary Habits Stratified by Fish Consumption

In the study groups, 32 women consumed between 0 and 2 servings of fish per week, whereas 65 women consumed between 3 and 4 servings of fish per week in their usual diet. Increased fish consumption (exceeding the recommended ranges) was observed in 46 (5 to 7 servings per week) and 8 (8 to 14 servings per week) women (Table 1). Anthropometric and biological values of the studied women, stratified by fish consumption, are shown in Table 2.

Nutrient intake was also studied, and the results are shown in Table 3. Dietary habits of the studied sample, stratified by fish consumption, are shown in Table 4.

**Figure 1:**
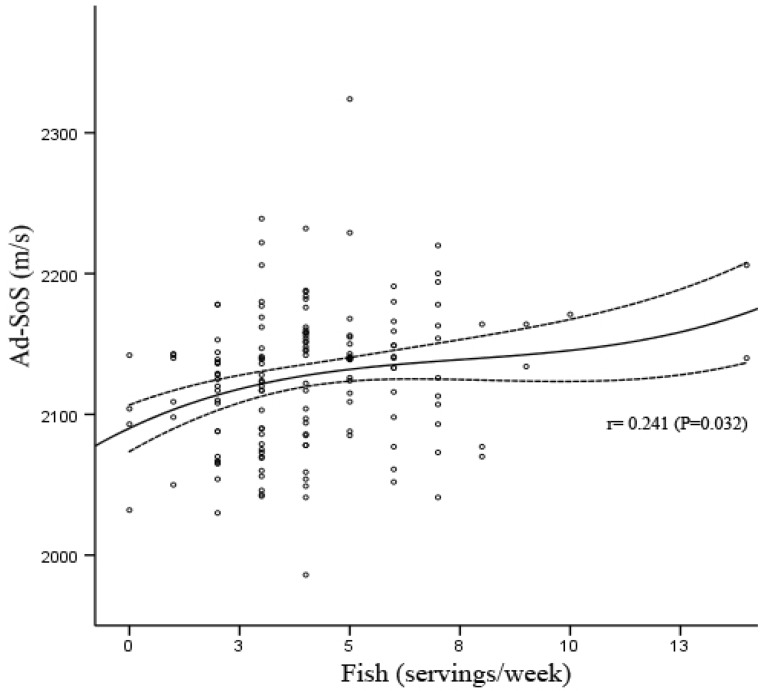
Best fit relationship between Ad-SoS (m/s) and fish consumption (servings/week) using cubic regression model with 95% confidence intervals.

Fish consumption showed a significant association with bone mass at the measured site among the different groups studied (Figure 1). The low number of subjects (*n* = 8) who consumed more than 8 servings of fish per week severely limited the ability of the study to detect the relationship of higher intake of fish with bone mass and other studied parameters. Higher Ad-SoS measurements (*P* < 0.05) in the fish intake group of 5 to 7 servings per week compared to the 0–2 servings per week group was observed (Table 5). Significant differences between the 8–14 servings/week group and the 0–2 and 3–4 servings/week groups were also observed in the intake of vitamin D and Zn, nutrients that are associated with the consumption of fish (Table 5). Intakes of other nutrients were also higher in the groups that consumed more servings of fish per week (proteins, carbohydrates, F, Cu and Se) (*P* < 0.05 in all cases).

There were no differences between the studied groups in age, weight, height, BMI or Ca intake (*P* > 0.05 in all cases). 

**Table 5:** Fish consumption and significance levels of comparisons between groups.

	Fish (servings/week)			
	0–2 (*n* = 32)	3–4 (*n* = 65)	5–7 (*n* = 46)	8–14 (*n* = 8)
Ad-SoS (m/s)			*P* < 0.05 *vs.* (0–2)	
Vitamin D (IU/day)			*P* < 0.05 *vs.* (0–2) and (3–4)	
Zn (mg/day)				*P* < 0.05 *vs.* (0–2) and (3–4)
Proteins (g/day)			*P* < 0.05 *vs.* (0–2)	*P* < 0.05 *vs.* (0–2), (3–4) and (5–7)
Proteins/weight (g/kg)			*P* < 0.05 *vs.* (0–2)	*P* < 0.05 *vs.* (0–2) and (3–4)
Carbohydrates (g/day)				*P* < 0.05 *vs.* (0–2) and (5–7)
Kcal		*P* < 0.05 *vs.* (0–2)	*P* < 0.05 *vs.* (0–2)	*P* < 0.05 *vs.* (0–2)
F (μg/day)		*P* < 0.05 *vs.* (0–2)	*P* < 0.05 *vs.* (0–2)	*P* < 0.05 *vs.* (0–2), (3–4) and (5–7)
Cu (mg/day)			*P* < 0.05 *vs.* (0–2)	*P* < 0.05 *vs.* (0–2) and (3–4)
Se (μg/day)				*P* < 0.05 *vs.* (0–2)
Vegetables (servings per week)			*P* < 0.05 *vs.* (0–2)	*P* < 0.05 *vs.* (0–2)
Eggs (servings per week)				*P* < 0.05 *vs.* (0–2) and (3–4)
Wine (servings per week)				*P* < 0.05 *vs.* (3–4)

**Figure 2:**
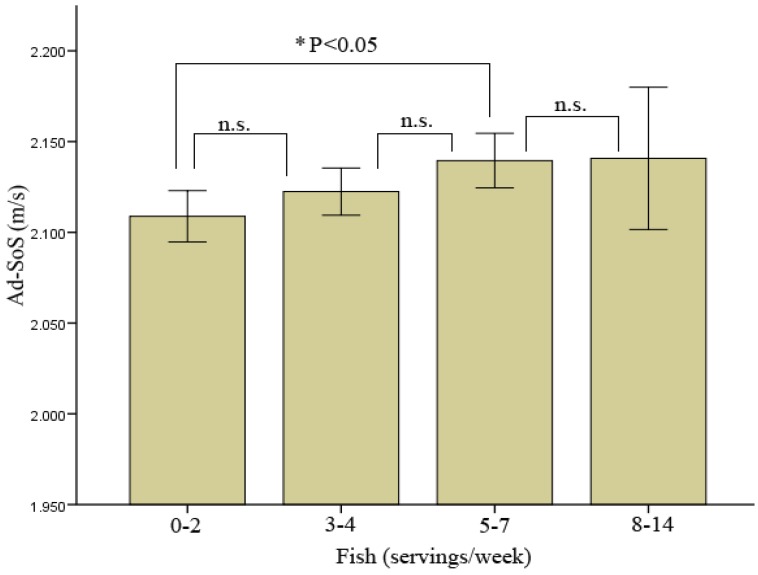
Association of fish intake on bone mass measured at the phalanges in premenopausal Spanish women. Values expressed as mean ± SD. * *P* < 0.05, n.s. (not significative).

Bivariate correlation analysis of all subjects showed a relation between the following variables and Ad-SoS measurements: weight (*r* = −0.164; *P* = 0.045), BMI (*r* = −0.282; *P* < 0.0001), Se intake (*r* = 0.168; *P* = 0.04) and fish consumption (*r* = 0.23; *P* = 0.005). The linear relations between Ad-SoS and weight, BMI and Se intake disappeared after adjustment for potential confounders (age, weight and height); the relation with fish consumption remained after adjustment for potential confounders, but the strength of the association decreased moderately (*r* = 0.227; *P* = 0.007). The cubic regression curve of fish consumption and Ad-SoS (*r* = 0.029; *P* < 0.001) is shown in Figure 2.

When the participants were aggregated in fish consumption groups, Carbohydrates (*r* = −0.957; *P* = 0.043) in the 0–2 servings/week group and fish consumption in the 3–4 servings/week group (*r* = 0.747; *P* = 0.033) remained as predictive variables after adjustment for potential confounders (age, weight and height). 

The main determinants of Ad-SoS were examined by multiple regression analysis. The variables entered in the model as independent predictors were age, weight, BMI, nutrient intakes and dietary habits. BMI and fish consumption were the variables that contributed significantly to explain Ad-SoS variance in premenopausal Spanish women (*β* = −8.482, *F* = 10.091, *P* = 0.001) and (*β* = 5.319, *F* = 10.091, *P* = 0.006), respectively.

## 4. Discussion

The fish consumption rate in Spain (43.2 kg/year per capita) is one of the highest in Europe (third, behind Portugal and Norway) and close to that of Japan, which has one of the highest rates in the world [18]. The fish consumption habits in our study are similar to those observed in other studies of Spanish women (4.17 *vs.* 4.20 servings/week) [19]. In this population-based cross-sectional study, we found that greater fish intake was associated with greater bone mass at the phalanges among premenopausal Spanish women. In general, our results are consistent with previous studies. It has been found that total body bone mineral density (BMD) correlates with seafood consumption in peri- and postmenopausal women (>250 g/week *vs.* <250 g/week; *P* < 0.01) [12]. Other authors compared the BMD in adult women (pre-, peri- and postmenopausal) living in a fishing village in Japan with age-matched subjects of urban origin and found that urban women consumed less fish and had decreased radial BMD [20]; a similar result was found by another study [21]. One investigation in Japan reported a lower risk of hip fracture associated with an increase in fish consumption [11], but this result was not confirmed by a different study [13]. 

In our sample, the positive association observed with a high intake of fish can be, in part, explained by the substitution of low-quality protein for fishprotein. In Spain, the ratio of fish to animal protein has been quantified at 20.7%, and the ratio of fish protein to total protein at 12.6%, which is one of the highest ratios in Europe [18] and the world, highlighting the importance of fish in the Spanish diet. Additionally, fish, especially oily fish, contain high concentrations of vitamin D and *n*-3 fatty acids. The mean value of vitamin D, a key nutrient for the maintenance of optimal bone mass, was 252.02 IU/day in the total sample and was higher in the group with an intake of 5–7 servings of fish per week, at 325.84 ± 246.79 IU/day. *N*-3 fatty acids were not quantified in our study, but the daily intake derived from fish in the Spanish diet has been calculated to be between 0.3 and 1.2 g [22].

Fish have been estimated to contribute up to 87% to the total dietary vitamin D intake in the Spanish adult population [1], and fish are also the primary source of vitamin D in adolescents [23]. Because the subjects who ate fish frequently (≥4 servings/week) had significantly higher levels of vitamin D than the subjects who ate fish 0–3 times/week, the frequency of fish consumption may be an important contributor to vitamin D intake in premenopausal women. However, we failed to demonstrate a significant correlation between fish consumption and vitamin D levels in our sample (*P* > 0.05). Fish may be a source of other trace elements, including selenium (Se), zinc (Zn) and copper (Cu), and deficiencies of these elements have been implicated in the increased risk of bone resorption through the inhibition of bone growth and the onset and progression of bone diseases, such as osteoporosis [24,25]. However, we did not find higher intakes of Zn and Se intakes in the 5–7 servings/week group *vs.* lower intakes groups (*P* > 0.05). Although Cu intake was higher in the 5–7 servings/week group *vs.* the 0–2 servings/week group, the intake (1.21 mg/day ± 1.39) was below the recommended intakes for Cu in the Spanish population. 

Moreover, fish have a rich content of long-chain *n*-3 fatty acids of eicosapentaenoic acid (EPA) and docosahexaenoic (DHA), and several studies have shown the beneficial effects of *n*-3 fatty acids on bone health in both humans and animals [9,26] by a wide range of mechanisms [8,27]. Recently, however, the consumption of marine *n*-3 fatty acids has been unexpectedly associated with a greater total fracture risk in postmenopausal women [28]. Although long-chain *n*-3 fatty acids may play an important role in the beneficial effect of fish on bone mass, but we were unable to accurately estimate the dietary intake of these fatty acids. Additionally, we did not have serum or blood cell samples to assess serum 25(OH)D, *n*-3 fatty acid or trace element concentrations. Further studies are needed to determine whether the positive association of fish intake with bone mass is mediated by higher intakes of vitamin D and *n*-3 fatty acids.

Studies of the association between protein intake and bone status report inconsistent results: with beneficial associations [29]; no association [17]; and adverse associations [30]. In the studied sample, the greater protein intake in relation to the Spanish and European Union RDAs is clearly significant, but no association with the bone mass was found. Therefore, our data do not support a negative association of protein with bone health, but findings in young women could be different from those in older women because bone mass is still accruing in young women [31]. Additionally other factors such as nutritional status may counteract the effects of a high protein intake [17].

A limitation of this cross-sectional study is that we could not establish temporality; thus, a causal relationship cannot be determined due to the unclear time sequence between the exposure and the outcome. 

## 5. Conclusions

The present cross-sectional study, which targeted a population of healthy premenopausal Spanish women, yielded the following findings: 

Fish consumption was positively associated with bone mass at the phalanges, measured with bone ultrasound, in Spanish premenopausal women. 

The mean vitamin D intake in the studied sample was highest in the group consuming 5–7 servings/week of fish. 

These findings suggest that increased fish consumption is helpful in maintaining adequate bone mass. Future studies should use analytic techniques to clarify the relationship of fish consumption with serum 25(OH)D, Zn and *n*-3 fatty acid concentrations. 
